# Academy of Stomatology

**Published:** 1901-01

**Authors:** 

**Affiliations:** Academy of Stomatology


### ACADEMY OF STOMATOLOGY.

A REGULAR monthly meeting of the Academy of Stomatology of Philadelphia was held at the rooms of the Academy, 1731 Chestnut Street, on the evening of June 26, 1900, the President, Dr. Lippincott, in the chair.

A paper entitled "Prophylaxis in Dentistry" was read by Dr. D. D. Smith, of Philadelphia.

(For Dr. Smith's paper, see Vol. XXI., page 805.)

Before the reading of the paper a recess was declared, during which Dr. Smith exhibited a number of patients whose mouths exemplified his ideas of prophylaxis.

### DISCUSSION.

*Dr. James Truman.*—I am heartily in accord with the whole paper. It seems to me that dentistry is drifting gradually in the direction of prophylaxis. There is no doubt but that in the future patients will be required to come regularly to the office to have their teeth attended to. I do not know that there has been anything new presented in so far as cleansing the teeth is concerned, but that dentists have been careless in training their patients, and that they should recall them once in every month or two to have their teeth thoroughly gone over, has been brought out and insisted upon by the essayist. I have not the least doubt that much pyorrhœa, gingivitis, and other disagreeable conditions that we have to contend with would be obviated by this procedure. The younger progressive members of the profession had better work in the direction of prophylaxis, for it is certain to be of increasing importance. Whether it will stop decay or not time alone will tell.

*Dr. Darby.*—Perhaps I cannot say anything that would be more valuable and convincing than to testify to what I saw a few weeks ago in Dr. Smith's office. He very kindly and courteously invited me there, and had present about a dozen of his patients to illustrate this method of treatment, and when I tell you it was a revelation and an inspiration to me, I only express about half of what I then felt and feel to-night. I have never seen teeth so absolutely clean and polished upon every surface as those that I saw there. I do not recall a single instance of a mouth with any deposit, with the exception of a young man who smoked pretty constantly. The teeth seemed to present a peculiar polished surface, which I could only liken to that of a ball polished on a lathe. The whole character of the tooth seemed changed. You have all seen in the mouths of some of your patients, where the teeth have been the best, a peculiar polished look which you have not seen in the mouth of your average patient, a peculiar ivory-like look of hardness, if I may so express it, throughout the whole enamel. I saw that in young girls, in middle-aged persons, and in two quite advanced in life in Dr. Smith's office; and when I went back to my own practice it made me very careful about the methods of my patients, and I preached to them as I never preached before. I have said more discourteous things than ever before. I have said to Harry or Jim, "Your teeth are dirty," while they might not have been worse than they ever were at any other time. I have said, "Your teeth are not clean; you do not take care of your teeth." Since I saw those patients I have taken better care of my own teeth, and the influence on my patients, indirectly through me, has been for good.

When Dr. Smith says he prevents decay, I believe he does. I

believe that if we remove the bacteria from the surface of the teeth by polishing, as he does, the teeth will not decay. There was only one point I could not understand, and that was how his patients could have teeth so clean upon the proximal, labial, and lingual surfaces without the use of floss silk; but their teeth were free from deposit, *débris,* or anything else, and the gums seemed to cling to the teeth as though they were part of them.

I simply want to indorse most heartily what I saw myself, and if the recommendations can be carried out *in toto* teeth will not decay and there will be very little use for the dentist, except to polish teeth.

*Dr. Kirk.*—I am very glad to add my few words of testimony to what Dr. Darby has already outlined to you of the wonderful character of the exhibit made by Dr. Smith in the mouths of his patients which he so kindly gave us an opportunity of examining. I do not think that I can add anything to what Dr. Darby has said. He has described the general principles of these methods, the peculiar appearance of the polished surface of the teeth, the character of the structure of the tooth, and the character of the mucous membrane and the gum. The mouth was free from the irritating effects of the bacteria, and we know infection may manifest itself in a very great many ways.

The thing that strikes me most forcibly about what Dr. Smith has accomplished is this: He seems to be the only man who has actually accepted at full face value the statements and results of investigations of those who hold to the idea that caries of the teeth is the result of the environment of the teeth. Its cause is external to the teeth. It seems to me a very rational conclusion that if the carious process is produced by these bacteria the one way to avoid caries is to get rid of them. Certainly there could be no bacterial plaque form upon the surfaces of teeth that have been treated by this method of Dr. Smith. It seems to me that he has gotten down to the tooth-structure. There is none of that slippery, greasy appearance and feeling commonly found in the mouths of those who take only ordinarily good care of the teeth. What we call ordinarily good care is not sufficient to protect dentures from carious influences. It must be extraordinarily good care, such as has been shown. The combination of an appreciation of cause and the effective operation of correctives has produced results for Dr. Smith which he can offer as evidence of the success of his application.

I must confess that I am not wholly satisfied with the exhibit made by Dr. Smith in one particular. I think we should have the record of a longer period of test of this matter before we decide whether the particular methods he suggests are the best for the accomplishment of his purpose. He has shown the way out. He may, in the course of time modify his treatment very much. So long as we have bacteria on the teeth and the lack of resistance in the tooth-structure, so long will we have caries.

The principle is all right. It is a question whether the tooth-polishing, carried over years of time, may not produce some modification of structure, which would indicate that it would be better to modify this method by less abrasive powders or employ some other method. I do not say that it will be so, but only raise the question. It will probably be a long time before we come to the final judgment of the utility of this particular method. I have nothing but words of commendation and approval.

*Dr. Darby.*—I would be very glad if Dr. Smith would tell us what specific instructions he gives his patients after seeing them, or if he sees his patients every month.

*Dr. Smith.*—I give the patients no specific instructions beyond what I have always done, that is, to clean the teeth carefully with brush and tooth-powder, which, in so far as possible, I select, and with all the younger patients I insist on their using fine salt, once a week, as a dentifrice. There are others to whom I do not give any particular instructions. I do not ask them to silk out the teeth. I have heard patients say they take half an hour of an evening "silking" their teeth; instead of this, if a quill toothpick were used after each meal and the teeth carefully cleansed with brush and dentifrice once a day, it would be far better for the teeth. After the surface of the enamel has been polished two or three times, brushing is much more helpful to the teeth than could possibly be the case prior to their having been polished; in other words, a very little effort on the part of the patient will put the teeth in a good condition. I would be very glad for any suggestions from any source whatever that would assist in the betterment of methods; but after having used this method for six years on a few patients, and in general practice for two years, I believe the methods will not be greatly modified. I consider it an impossibility for any patient to take such care of the teeth as will keep them absolutely free from bacterial accumulations. It requires outside interference, and that outside interference consists of an intelligent removal by the operator of the accumulations by the methods indicated.

Dr. Darby spoke of silking out between the teeth. It is an operation which nature never designed should be performed in the mouth at all; the teeth are placed in apposition, and all that is required is that the proximal surfaces shall be kept clean and polished. The uncultured colored people of the South, in their original condition of slavery, were compelled to eat bacon, pork, and cracked corn. You will find the teeth of the field-hands generally in good condition; they never silk out their teeth. Nature has placed teeth alongside of each other, and never designed that anything should go in between them. All surfaces of a tooth ought to be polished just as the occlusal surfaces are polished, by mastication. The patient cannot do this, the dentist has not done it, and the result has been, as you know, unlimited decay. I do not know that it will be necessary to continue this practice throughout life. I do know that it is absolutely essential that it should be done in early life, during the formative period, and unless it is done you will find, in every instance, a pathological condition of the child's mouth. It is the business, it seems to me, of the dentist to make good teeth, not by going back to the mother's breast and attempting to control the food of the child, but by preventing them from being contaminated by the external agencies which cause their decay. That, however, is not the only benefit derived from the polishing as advocated. The irritation which this process gives to the outside of the teeth causes positive stimulation of the pulp and the deposition in these teeth of better dentinal material, and this is where the chief benefit comes from the care of the teeth in childhood. You will say that they ought to get this in the masticating process. So they should to a degree; but with the present methods of preparing food, and the consequent lack of use of the teeth, they never get sufficient irritation from mastication to give that stimulus which causes the pulp to deposit a good constructive tooth-material. This irritation is supplied to some extent in brushing, and at the same time there is fulfilled the object of divesting the external surface of the teeth of those agents which cause their decay. I esteem this stimulus to better tooth-building the strongest point in connection with this practice. If there should be any modification, as suggested by Dr. Kirk, it will be a partial abandonment of the process later on in life. I do not know that it will be necessary to continue it, but I do know this: I have yet to find the first case of a young adult, middle-aged, or old person, where the benefits accruing to the tooth have not been marked after even a few treatments. The life-forces of the tooth work, as the result of this treatment, to produce better tooth-material.

I wish to thank you, gentleman, for your courteous and very kind attention; on account of the extreme heat I must leave the subject with you for any further consideration that you choose to give to it. I now bid you good-night.

*Dr. Cryer.*—I would move a vote of thanks to Dr. Smith for his valuable paper and subsequent talk.

Adopted.

*Dr. William Trueman.*—In looking at the cases presented and listening to the doctor's remarks, I am impressed with the claims for what will take place within two or three months from a constant irritation, and question whether in the end it will not be disastrous. A case of pyorrhœa has been under my observation where I have shown that the usefulness of the teeth has been curtailed by that process. Had milder treatment been pursued and less attention paid to irritation, the teeth would have been in better condition. The best germicide we can use in the mouth, one that we can always depend on and one that can possibly do no harm, is perfectly healthy saliva. Dr. Kirk published a paper a little while ago, giving his observations at a deaf and dumb asylum. I think he probably lost sight of it when speaking. At the time it was published he gave the dietary of that institution. He brought out the fact that it was not the dietary alone, but the regular lives led that gave the inmates hard teeth. In that case a more healthy condition of the system was brought about. If we could induce in all persons a perfectly healthy condition, we would all die of old age. With the method of thoroughly cleansing the teeth by rubbing and scrubbing them to keep them free from deposits, it is a question whether we do not do more harm than good. There is a happy medium in all these things, and those who strike it derive better results. I think that in five years from now the doctor will think very differently from what he does now, but we are very thankful to him for bringing up the question, and while some may not accept it, some will, and experience will prove or disprove it.

*Dr. James Truman.*—I should like to add one more word. I do not exactly like Dr. Smith's idea of the contraindication of floss silk. I cannot understand why the silk passed between proximal surfaces is not the very best cleanser that can be used for these surfaces. Again, the so-called plaques have never been scientifically demonstrated to be acid producers, and yet we hear and talk of those matters as though the whole thing were settled. Those gentlemen who so determinedly insist that the bacteria in these plaques are acid producers had better read Goadby on that same point in the *British Journal of Dental Science.*

*Dr. Roberts.*—The argument which Dr. Smith advances, that nature did not intend silk to be used, can be applied to all other forms of cleansing teeth that he suggested. If nature did not intend that floss silk should be used, she did not intend that teeth should be brushed or that pumice-stone should be used. If you use the argument for one thing, why not for another analogous thing?

I think there is no germicide which can be used in the mouth which is liable to be held there sufficiently long to destroy the bacteria, and that while the germicide may not do any harm, it will not cleanse the teeth; neither will it prevent decay, unless there is other instrumentation with that, and the instrumentation is more valuable, in my opinion, than the germicide.

*Dr. Jack.*—I may state that my views are generally in accord with Dr. Smith's statement; that is, my experience has borne out the fact that just in proportion as I have been able to get young children or my young patients to take better care of the teeth have I been successful in securing a good condition. I remember the instance of one boy in my practice: he had a good many complications, and was eighteen years of age. I had cared for his teeth probably two or three times a year, each time carefully cleansing them myself, both within and without and between the teeth, and I endeavored to teach him how to care for his teeth. At last I trained him to take reasonably good care, and after a couple of interviews I found nothing to be done; at the last of such interviews he got up out of the chair with the expression, "It pays to take care of your teeth." I remember another instance of a young lad in whom I had filled a large number of proximal cavities in bicuspids and molars, and upon whom I could not impress the necessity for keeping his teeth clean. He was about going to Europe at one time, and I put a large number of fillings in his teeth, some extending to the cervical margin. I stated to him the consequences caused by his own neglect, and that if he would take proper care of his teeth it would limit the caries. At the same time I put his mouth into good condition. He went to Europe, took the lesson to heart, and came back to me after two years of absence, and I could not find caries in his teeth. Whether that was partly due to better hygienic conditions, incident to his travels in Europe, I cannot answer. Certainly that was the result, and the boy has had since very little decay in the teeth. He has a brother, on the other hand, whose teeth were somewhat softer. I could never get him to take proper care, and he has been suffering considerably with proximal caries.

I examined at one time some years since the mouths of Indian girls who were at the Lincoln Institute, who had been brought from various parts of the Western reservations, from the borders of Canada down to New Mexico, and those Indian tribes who possessed the best teeth were the Sioux Indians. I may state here that one single cross of the white man on the middle and southern tribes of Indians always showed a marked increase of caries over the other Indians. In the first cross between the white man and Sioux, the open sulci were true; when it came to the second cross, caries began to appear. Some of the pure races belonging to New Mexico and other Southern races presented caries of the teeth, and in the real Indian, whereas the Sioux presented none. Here were conditions exactly alike in both cases,—the Southern Indian lived as the Northern Indian did. My only explanation is this: The Sioux, living in the higher northern latitudes, were subject to greater vicissitudes of climate, and also probably other conditions that led to the survival only of the high constitutional conditions, whereas the Southern Indians lived under better conditions, under better climate and less physical stress,—that is, of inherently different conditions,—and grew up to maturity. It certainly showed that the question of environment did not induce decay of the tooth, because we might infer that the habits of the Sioux were no different from the habits of the Southern Indians.

At some time or another I will present a tabulation before this society. I have meant to do it from time to time, but have waited until I have had an opportunity to examine a larger number of Indians, so as to make the matter more comprehensive and the results more definite in the conclusions that might be taken from them.

*Dr. Hickman.*—I am disappointed that some one has not spoken of the white spots that will appear on the teeth, that Dr. Smith told us should be removed. I have several patients with white spots on the labial surface of the front teeth, and I would like to know if any one knows how to get rid of them, or if this method is certain to effect it.

*Dr. Schamberg.*—I am very sorry that this paper did not extend to the prophylactic treatment for the prevention of diseases affecting the soft and hard tissues, which are likely to follow certain diseases, inasmuch as prophylaxis in dentistry should include that form of treatment. I must say that I believe that prophylaxis after disease, such as measles, scarlet fever, small-pox, and diphtheria, is frequently neglected, and I take this opportunity of exhibiting a few photographs that I have of cases of gangrenous stomatitis, which, though it may seem far-fetched, I think is generally probably due to the fact that prophylaxis is not thoroughly practised in these cases. I may state that of these three cases of gangrenous stomatitis, every one of them followed measles. However, two of them were suffering or convalescing from other diseases. One was convalescing from measles; one had pneumonia after measles, and the other had diphtheria. The thought came to my mind of the relationship between the prophylaxis in this direction and the prophylaxis of the teeth, through a case that appeared at the surgical clinic of the Polyclinic Hospital for treatment, where we removed a necrosed area of bone, involving the two superior deciduous central incisors and the partially formed permanent central incisors. It simply shows the necessity for the use of germicidal agents in the mouth after such diseases as measles, scarlet fever, diphtheria, and small-pox. We know that measles is quite prevalent, even among the better classes, although small-pox is not so frequently found, and scarlet fever is of rather frequent occurrence. Practitioners fail to remember that the eruptions in this disease are frequently found in the mouth antedating the eruption in other parts of the body. I will pass around these photographs, one showing the condition as it started, and another showing it as it advanced. In gangrenous stomatitis the disease is fatal in perhaps eighty or ninety per cent. of cases, and therefore it shows that the only way to combat the disease is to prevent it, and not to attempt to treat it after it has once begun its ravages, for unless you remove almost the entire part, just as you would in any other gangrenous area, you cannot hope to have any results whatever. The patients are usually so weakened by the condition that they succumb to the operation.

Adjourned.

OTTO E. INGLIS,
*Editor Academy of Stomatology.*

